# Electronic medical record‐verified hepatitis C virus screening in a large health system

**DOI:** 10.1002/cam4.2247

**Published:** 2019-06-21

**Authors:** Monica L. Kasting, Anna R. Giuliano, Richard R. Reich, Linh M. Duong, Julie Rathwell, Richard G. Roetzheim, Susan T. Vadaparampil

**Affiliations:** ^1^ Department of Health and Kinesiology Purdue University West Lafayette Indiana; ^2^ Department of Cancer Epidemiology Moffitt Cancer Center Tampa Florida; ^3^ Center for Immunization and Infection Research in Cancer Moffitt Cancer Center Tampa Florida; ^4^ Department of Biostatistics and Bioinformatics Moffitt Cancer Center Tampa Florida; ^5^ Department of Health Outcomes and Behavior Moffitt Cancer Center Tampa Florida; ^6^ Department of Epidemiology and Biostatistics University of South Florida Tampa Florida; ^7^ Department of Family Medicine University of South Florida Tampa Florida

**Keywords:** cancer screening, electronic medical records, health care utilization, hepatitis C, hepatocellular carcinoma, viral hepatitis

## Abstract

**Background:**

Baby boomers are at increased risk for hepatitis C virus (HCV) infection and related cancer; therefore, one‐time HCV screening is recommended.

**Methods:**

To assess prevalence of, and factors associated with providers ordering HCV screening, we examined a retrospective cohort of electronic medical records for patient visits from 01 August 2015 until 31 July 2017 in a large health system. HCV screening ordered was examined by patient age, gender, race/ethnicity, provider specialty, and number of clinical visits, stratified by birth cohort: born ≤1945, 1945‐1965 (baby boomers), 1966‐1985, and ≥1985. Multivariable regression identified factors independently associated with HCV screening ordered among average risk baby boomers.

**Results:**

A total of 65 114 patients ages ≥18 years were evaluated. Among baby boomers HCV screening test order increased threefold between the two study years (4.0%‐12.9%). Odds of screening test ordered were significantly higher for non‐Hispanic Blacks (multivariable adjusted odds ratio [aOR]=1.36; 95% CI = 1.19‐1.55), males (aOR = 1.44; 95% CI = 1.33‐1.57), and having a clinic visit with a primary care provider alone or with specialty care (aOR = 3.25‐4.16). Medicare (aOR = 0.89; 95% CI = 0.80‐0.99), Medicaid (aOR 0.89; 95% CI 0.80‐0.99), and an unknown provider type (aOR = 0.16; 95% CI = 0.08‐0.33), were associated with lower odds of screening tests ordered.

**Conclusions:**

While the proportion of baby boomers with an HCV screening test ordered increased during the study, the rate of screening remains far below national goals. Data from this study indicate that providers are not ordering HCV screening universally for all of their baby boomer patients. Continued efforts to increase HCV screening are needed to reduce the incidence of HCV‐related morbidity and mortality.

## 1 INTRODUCTION

Liver cancer is 1 of only 3 cancers that have increased in incidence and mortality in the United States over the last decade.^1^ Approximately half of all liver cancers in the US are caused by hepatitis C virus (HCV) infection.^2^, ^3^ Early detection and treatment of chronic HCV infection can reduce cancer incidence by 75%,^2^ but ~50%‐75% of those with HCV are unaware of their infection.^4^, ^5^ Individuals born between 1945 and 1965 (ie, baby boomers) have nearly 5 times the prevalence of HCV infection compared to other birth cohorts.^6^ Almost half of those infected report no risk behaviors and are unlikely to be identified without an age‐targeted screening program.^7^ In Florida, approximately half of chronic HCV cases are in individuals over 50 years old.^8^

Therefore, in 2012, the Centers for Disease Control and Prevention (CDC) augmented their risk‐based HCV screening recommendation to include 1‐time universal screening for everyone born 1945‐1965,^7^ and the United States Preventive Services Task Force (USPSTF) issued a similar statement the following year.^9^ However, nationally representative data from 2015 indicate less than 13% of baby boomers had ever been screened and rates have not meaningfully increased over time since the recommendation was issued in 2012.^10^ Recent advances, such as rapid finger stick HCV testing and direct acting antivirals (DAAs), with cure rates over 95% for treating HCV infection, lead to optimism that HCV related diseases can be reduced. However, achieving this goal is dependent on providers adopting public health recommendations to screen eligible patients for HCV infection.^11^, ^12^, ^13^, ^14^ Therefore, the primary aim of this study was to assess the prevalence of provider HCV screening orders using a large health system in Florida to establish a baseline from which to monitor future intervention effects. An additional aim was to assess factors associated with HCV screening tests ordered.

## 2 MATERIALS AND METHODS

### 2.1 USF health system and EMR

We conducted a retrospective cohort study examining electronic medical records (EMR) for patient visits during the 2‐year period between August 1, 2015 and July 31, 2017 within the University of South Florida (USF) Health system. Our outcome of interest was whether a patient had HCV screening ordered by their provider and the exposure of interest was birth cohort. USF Health is the largest physician group in the Tampa Bay area with 400 practicing physicians and 506 897 outpatient visits in 2015. The practice has used an EMR for more than 10 years, and has used Epic since August, 2015. As Epic compiles comprehensive health services data, which facilitates evaluation of procedures such as HCV screening, we restricted this analysis to the 2‐year time period immediately following implementation of Epic at USF Health. Clinical data from 1 139 858 unique patients are kept in a searchable data warehouse that can be downloaded for health services research. HCV screening is included in the health maintenance tab in the EMR of all patients born between 1945 and 1965. This tab reminds physicians that all age‐appropriate patients are due for HCV screening. Only patients over 18 years of age were included in the analyses.

EMR data included information regarding the encounter type (eg, office visit, surgical consult, etc) and provider type (eg, physician, physician assistant, and nurse practitioner). Before applying our exclusion criteria, the entire beginning dataset for this study included more than 150 000 individual patients, 823 000 clinic visits, and 4 million procedure codes. Specifically, we focused on whether HCV screening was ordered by the provider. The study was approved by the Institutional Review Board at USF.

### 2.2 Clinical data extraction

**Figure 1:**
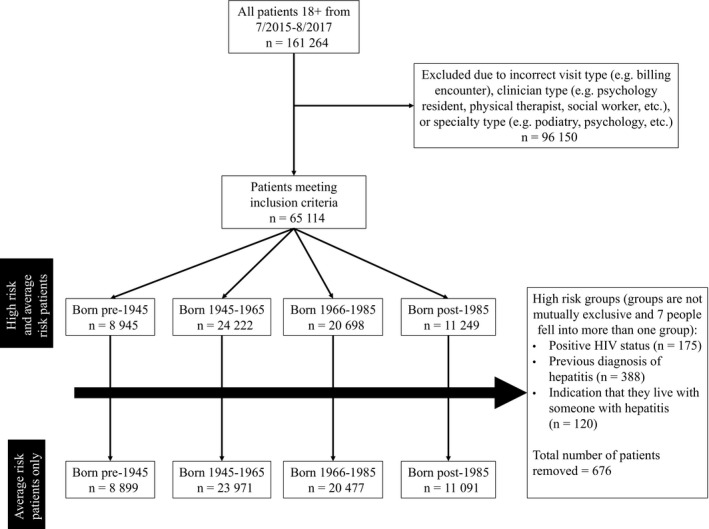
Flowchart of Participant Inclusion/Exclusion

We used the EMR data repository to identify all completed patient encounters from August 2015 through August 2017. Patients were stratified by birth cohort: those born before 1945, those born between 1945 and 1965, those born between 1966 and 1985, and those born after 1985. Because the recommendation for universal screening is only for the 1945‐1965 birth cohort; including the other cohorts provides a point of comparison to assess whether the 1945‐1965 cohort demonstrates an increase in increased relative to older and younger birth cohorts. Patients who only had visit types (eg, billing encounter), clinician types (eg Psychology Resident), or specialty types (eg, Podiatry) not relevant to primary care visits were eliminated from the dataset. We included patients who saw either a primary care provider (eg, family medicine, general internal medicine) or a relevant specialist (eg. gastroenterologist, infectious disease specialist, etc). To assess HCV screening we used procedure codes included in the EMR. For a step‐by‐step flowchart illustrating study inclusion and exclusion, please see Figure 1.

### 2.3 Variable description

All HCV antibody screening tests ordered during any patient encounter in the 2‐year study period were identified. This was coded as a binary yes/no variable. Factors examined for association with HCV test ordering among baby boomer patients included: patient age, race/ethnicity, sex, and preferred language. We also assessed healthcare factors including the patient's health insurance coverage, number of primary care visits during the 2‐year study period, and the number of visits to a specialist during the 2‐year study period. Lastly, we assessed factors that typically result in higher percentages of HCV screening including positive HIV status, previous diagnosis of hepatitis, and an indication that they live with someone with hepatitis.^7^

### 2.4 Data analyses

First we described demographic, medical, and risk variables (eg, lives with someone with hepatitis) for the entire sample stratified by birth cohort. We then assessed the percentage and 95% confidence interval of patients who had an HCV screening test ordered stratified by study year and birth cohort. To assess HCV screening among average‐risk patients, we restricted the analysis to patients that had no established HCV or liver disease risk factors other than age (ie, HIV positive, history of liver cancer, history of hepatitis, lived with someone with hepatitis), and assessed frequency of HCV screening test ordered by year and birth cohort. We further examined frequency of HCV screening test ordered by selected demographic characteristics among average risk baby boomers. Finally, to identify factors independently associated with HCV screening test orders among average risk baby boomer patients, we conducted multiple variable logistic regression analysis. A significance level of 0.05 was utilized and all statistical analyses were conducted with SAS, version 9.4.

## 3 RESULTS

### 3.1 Study Population

The final analytic sample consisted of 8945 patients born before 1945 (mean age = 77.9; SD = 5.6), 24 222 baby boomers (mean age = 60.2; SD = 5.9), 20 698 patients born between 1966 and 1985 (mean age = 39.6; SD = 5.9), and 11 249 patients born after 1985 (mean age = 24.3; SD = 3.6). There were more females than males in every birth cohort, with the youngest age group having the highest proportion of females (74.7% female). Approximately 95% of the sample indicated their language preference was English, regardless of age. The majority of patients in the oldest birth cohort had Medicare insurance coverage (88.4%) compared to private insurance in the other three younger birth cohorts. For a full description of the sample, see Table 1.

**Table 1:** Socio‐demographic characteristics of patients in the University of South Florida Health System, 2015‐2017^a^

	Born pre‐1945 (n = 8945)	Born 1945‐1965 (n = 24 222)	Born 1966‐1985 (n = 20 698)	Born post‐1985 (n = 11 249)
	% (95% CI)	% (95% CI)	% (95% CI)	% (95% CI)
Age [Mean (SD)]	77.9 (5.6)	60.2 (5.9)	39.6 (5.9)	24.3 (3.6)
Race/ethnicity				
Non‐Hispanic White	75.0 (74.1‐75.9)	68.1 (67‐6‐68.7)	56.5 (55.8‐57.1)	55.1 (54.2‐56.0)
Non‐Hispanic Black	5.1 (4.6‐5.5)	9.8 (9.4‐10.2)	14.2 (13.7‐14.6)	14.8 (14.1‐15.5)
Non‐Hispanic Asian	1.4 (1.1‐1.6)	1.8 (1.6‐2.0)	3.8 (3.5‐4.0)	3.8 (3.4‐4.1)
Non‐Hispanic Other	12.2 (11.5‐12.9)	12.9 (11.5‐12.3)	12.3 (11.8‐12.7)	11.7 (11.1‐12.3)
Hispanic	6.5 (6.0‐7.0)	8.4 (8.1‐8.8)	13.3 (12.9‐13.8)	14.6 (13.9‐15.2)
Sex				
Male	41.6 (40.6‐42.6)	37.5 (36.9‐38.1)	28.3 (27.7‐28.9)	25.3 (24.5‐26.2)
Female	58.4 (57.4‐59.4)	62.5 (61.9‐63.1)	71.7 (71.1‐72.3)	74.7 (73.8‐75.5)
Language				
English	94.7 (94.2‐95.1)	95.1 (94.9‐95.4)	95.2 (94.9‐95.5)	96.7 (96.3‐97.0)
Spanish	2.7 (2.4‐3.0)	2.4 (2.2‐2.6)	2.2 (2.0‐2.4)	1.1 (0.9‐1.3)
Other	0.7 (0.5‐0.8)	0.6 (0.5‐0.7)	0.7 (0.6‐0.9)	0.7 (0.5‐0.9)
Don't know/Refused/Not ascertained	2.0 (1.7‐2.3)	1.9 (1.7‐2.0)	1.9 (1.7‐2.1)	1.5 (1.3‐1.8)
Health insurance coverage				
Military	0.4 (0.3‐0.5)	3.3 (3.1‐3.5)	4.2 (3.9‐4.5)	3.7 (3.3‐4.0)
Private	4.5 (4.1‐5.0)	55.2 (54.5‐55.8)	76.5 (75.9‐77.1)	78.6 (77.8‐79.4)
Medicaid	0.9 (0.7‐1.1)	4.3 (4.1‐4.6)	7.8 (7.5‐8.2)	11.2 (10.7‐11.8)
Medicare	88.4 (87.8‐89.1)	33.5 (32.9‐34.1)	9.1 (8.8‐9.5)	4.1 (3.8‐4.5)
Medicare supplement	4.9 (4.5‐5.4)	2.4 (2.2‐2.6)	0.3 (0.2‐0.3)	0.1 (0‐0.1)
Other	0.8 (0.6‐1.0)	1.3 (1.2‐1.5)	2.1 (1.9‐2.3)	2.3 (2.1‐2.6)
Primary care visits [Mean (SD)]	3.3 (6.3)	2.4 (4.5)	2.4 (3.6)	2.2 (3.3)
Specialty visits [Mean (SD)]	0.9 (2.1)	0.8 (2.1)	0.5 (1.6)	0.4 (1.6)
Risk factors				
HIV	0.1 (0‐0.2)	0.3 (0.2‐0.4)	0.3 (0.2‐0.3)	0.3 (0.2‐0.4)
Hepatitis	0.3 (0.2‐0.4)	0.4 (0.4‐0.5)	0.4 (0.3‐0.5)	0.7 (0.6‐0.9)
Lived with someone with hepatitis	0.1 (0‐0.1)	0.1 (0.1‐0.2)	0.1 (0.1‐0.2)	0.2 (0.1‐0.3)

a This table represents the entire study population, including high‐risk patients

### 3.2 Frequency of HCV screening test orders

**Figure 2:**
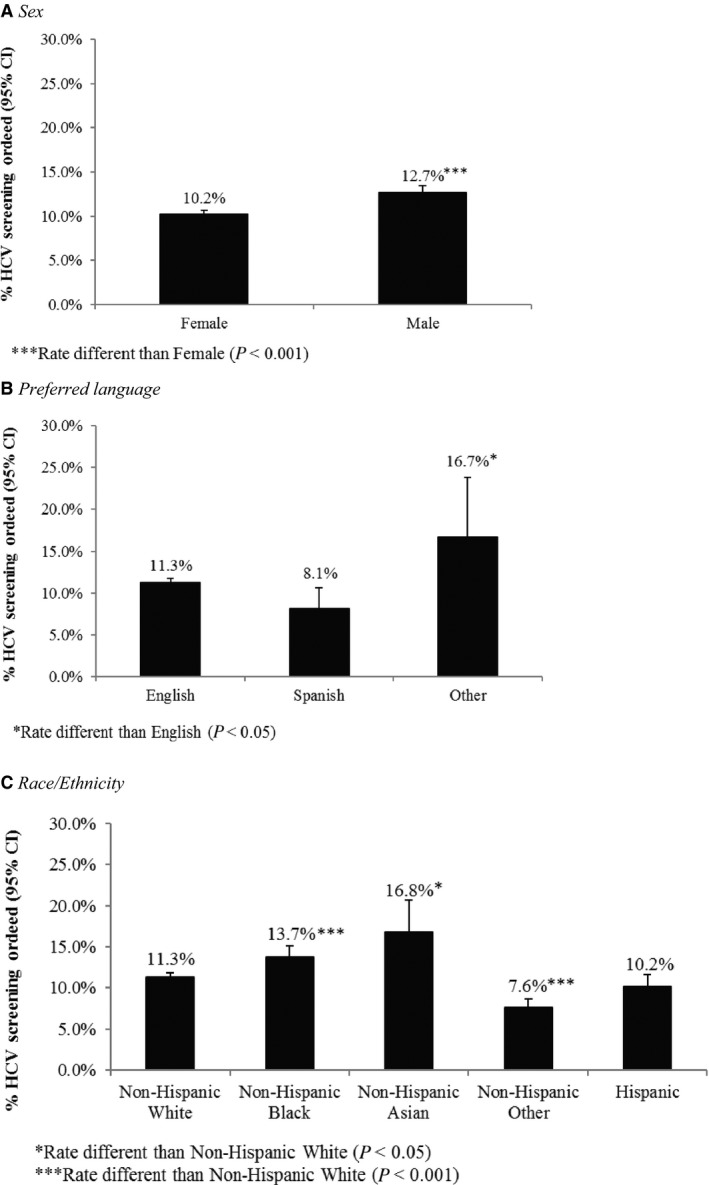
Differences in Hepatitis C Virus Screening Orders from 2015 to 2017 by Demographic Characteristics among Average Risk Baby Boomers

**Figure 3:**
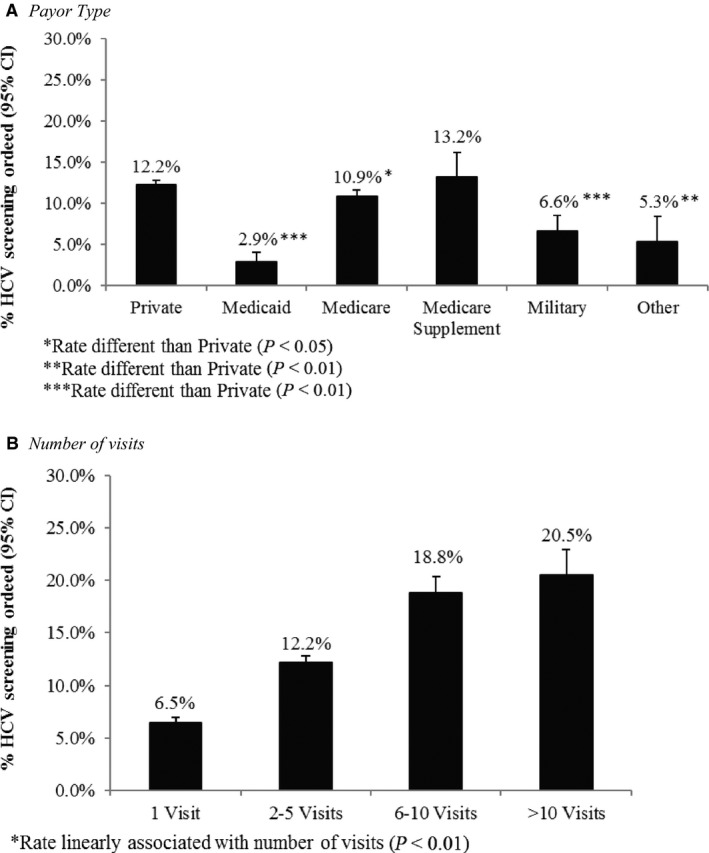
Differences in Hepatitis C Virus Screening Orders from 2015 to 2017 by Healthcare Characteristics among Average Risk Baby Boomers

The proportion of patients receiving an order for an HCV screening test significantly increased from year 1 to year 2 for those born before 1945 (*P* < 0.0001), did not change among those born 1966‐1985 (*P* = 0.08), and significantly decreased among those born after 1985 (*P* < 0.0001). The proportion of baby boomers receiving an order for an HCV screening test significantly increased from 4.0% in the first year of the study period to 12.9% in the second year of the study period (>threefold increase;* P* < 0.0001). When we restricted the analyses to only include average risk patients, the proportion with HCV screening tests ordered was comparable to the results in the overall population, possibly due to the small number of high risk patients (Table 2). Percentage of average risk baby boomers receiving an order for HCV screening by selected demographic characteristics (sex, preferred language, and race/ethnicity) are presented in Figure 2A‐C. HCV screening test ordered was highest for males (12.7% vs. 10.2%), those whose preferred language was something other than English or Spanish (16.7% vs. 11.3% [English] and 8.1% [Spanish]), and non‐Hispanic Asians (16.8% vs. 11.3% [non‐Hispanic White], 13.7% [non‐Hispanic Black], 7.6% [non‐Hispanic Other], and 10.2% [Hispanic]). The percentage of average risk baby boomers receiving an order for HCV screening by relevant healthcare characteristics (ie, payor type and number of clinic visits) is presented in Figure 3A,B. HCV screening test ordered was highest for those with a Medicare supplement (13.2% vs. 12.2% [private insurance], 2.9% [Medicaid], 10.9% [Medicare], 6.6% [military insurance], and 5.3% [other insurance]) and those who had more than 10 visits to a provider during the study period (20.5% vs. 6.5% [1 visit], 12.2% [2‐5 visits], 18.8% [6‐10 visits]).

**Table 2:** Hepatitis C virus screening ordered by year and birth cohort from 2015 to 2017

	Born pre‐1945 (n = 8945)		Born 1945‐1965 (n = 24 222)		Born 1966‐1985 (n = 20 698)		Born post‐1985 (n = 11 249)	
	2015‐2016 (n = 6314)	2016‐2017 (n = 6376)	2015‐2016 (n = 16 362)	2016‐2017 (n = 16 971)	2015‐2016 (n = 13 246)	2016‐2017 (n = 13 501)	2015‐2016 (n = 6424)	2016‐2017 (n = 7428)
All patients								
HCV screening ordered % (95% CI)	0.8 (0.6‐1.0)	1.8 (1.5‐2.2)	4.0 (3.7‐4.3)	12.9 (12.4‐13.4)	3.5 (3.2‐3.8)	3.9 (3.6‐4.2)	6.4 (5.8‐7.0)	4.3 (3.8‐4.7)
High‐risk patients removed								
HCV Screening Ordered % (95% CI)	0.8 (0.6‐1.0)	1.8 (1.5‐2.2)	3.9 (3.6‐4.2)	12.7 (12.2‐13.2)	3.5 (3.1‐3.8)	3.8 (3.5‐4.2)	5.7 (5.7‐6.9)	4.1 (3.7‐4.6)

### 3.3 Factors independently associated with HCV screening test orders

Factors associated with HCV screening test orders among baby boomers in are reported in Table 3. In the multivariable model, Non‐Hispanic Asian race/ethnicity (aOR = 1.39; 95% CI = 1.05‐1.83; reference = Non‐Hispanic White), non‐Hispanic Black race/ethnicity (aOR = 1.36; 95% CI = 1.19‐1.55), male sex (aOR = 1.44; 95% CI = 1.33‐1.57), preferred language other than English or Spanish (aOR = 1.64; 95% CI = 1.02‐2.63; reference = English), increasing number of healthcare visits (aOR = 1.02; 95% CI = 1.02‐1.03), having a clinic visit with both a primary care provider and a specialist (aOR = 4.16; 95% CI = 3.47‐4.99; reference = specialist visit only), and having a clinic visit with a primary care provider only (aOR = 3.25; 95% CI = 2.84‐3.73), were associated with higher odds of having an HCV test ordered. Factors associated with lower odds of having an HCV test ordered include Non‐Hispanic Other ethnicity (aOR = 0.77; 95% CI = 0.66‐0.90); having insurance with Medicaid (aOR = 0.26; 95% CI = 0.18‐0.39; reference = private insurance), Medicare (aOR = 0.89; 95% CI = 0.80‐0.99), military (aOR = 0.57; 95% CI = 0.43‐0.77), or other type (aOR = 0.44; 95% CI = 0.26‐0.75); and an unknown provider type (aOR = 0.16; 95% CI = 0.08‐0.33). For results of the regression analyses of the other three birth cohorts, see Appendices [Link], [Link], [Link].

**Table 3:** Factors associated with hepatitis C virus screening orders for average risk baby boomers from 2015 to 2017

	Univariate OR (95% CI)	Multivariable aOR (95% CI)
Born 1945‐1965		
Characteristics		
Age (continuous, 5‐year increments)	1.00 (1.00‐1.01)	1.00 (1.00‐1.01)
Race/ethnicity		
Non‐Hispanic White (ref.)	‐	‐
Non‐Hispanic Black	**1.24 (1.09‐1.41)**	**1.36 (1.19‐1.55)**
Non‐Hispanic Asian	**1.57 (1.21‐2.04)**	**1.39 (1.05‐1.83)**
Non‐Hispanic Other	**0.65 (0.56‐0.75)**	**0.77 (0.66‐0.90)**
Hispanic	0.89 (0.76‐1.04)	1.05 (0.88‐1.24)
Sex		
Female (ref.)	‐	‐
Male	**1.28 (1.18‐1.39)**	**1.44 (1.33‐1.57)**
Language		
English (ref.)	‐	‐
Spanish	**0.69 (0.51‐0.94)**	0.92 (0.65‐1.30)
Other	**1.57 (1.01‐2.44)**	**1.64 (1.02‐2.63)**
Payor		
Private (ref.)	‐	‐
Medicaid	**0.21 (0.15‐0.31)**	**0.26 (0.18‐0.39)**
Medicare	**0.88 (0.80‐0.96)**	**0.89 (0.80‐0.99)**
Medicare supplement	1.09 (0.85‐1.39)	1.08 (0.83‐1.40)
Military	**0.50 (0.38‐0.67)**	**0.57 (0.43‐0.77)**
Other	**0.40 (0.25‐0.66)**	**0.44 (0.26‐0.75)**
Total number of healthcare visits	**1.04 (1.03‐1.05)**	**1.02 (1.02‐1.03)**
Type of visits during observation period (July 2015‐August 2017)		
Specialty care physicians only (ref.)	‐	‐
Primary care physicians only	**3.53 (3.09‐4.04)**	**3.25 (2.84‐3.73)**
Primary and specialty care physicians	**5.01 (4.20‐5.97)**	**4.16 (3.47‐4.99)**
Advanced practice professionals only	1.04 (0.88‐1.24)	0.97 (0.82‐1.16)
Unknown	**0.17 (0.08‐0.34)**	**0.16 (0.08‐0.33)**

The bolded values are significant at *p*<0.05

## 4 DISCUSSION

Patients in our study had similar percentages of HCV screening ordered as compared to self‐reported national data reporting rates of HCV screening.^10^ In both the EMR‐verified and the self‐reported national data, HCV screening was well below the CDC and USPSTF screening guidelines for universal screening of baby boomers.^7^, ^9^ Healthy People 2020 has a goal of increasing the number of people who are aware of their HCV infection from 53% to 60%^15^; this is unlikely to happen without additional intervention.

Several factors were associated with increased HCV test orders in our study. As in national samples, men had a higher proportion of having screening ordered than women.^10^ This could be for several reasons. One possibility is that physicians continue to screen their patients based on perceived risk, as opposed to universally screening all baby boomers. It may be that providers have the opinion that men engage in riskier behaviors than women.^16^ There is also the possibility that men are being screened as a result of abnormal bloodwork; men have a higher incidence of liver cancer, viral hepatitis, and are more likely to die from chronic liver disease than women.^17^, ^18^ However, research also indicates that post‐menopausal women have higher rates of fibrosis progression in chronic HCV infection than younger women.^19^ Therefore, physicians should be made aware that as women enter menopause, their rate of progression to liver disease will increase, emphasizing the importance that baby boomer women be screened for HCV.

Additionally, odds of having an HCV screening test ordered differed by insurance type. Patients with private insurance or a Medicare supplement had the highest odds of receiving a referral for HCV screening. In this patient population, individuals with military insurance had lower odds of screening. The lower odds of screening may be due to patients having received HCV screening at a VA facility in the past, resulting in that screening test not being captured in our dataset. Recent research shows that patients receiving care at a Veteran's Affairs (VA) Hospital have higher rates of HCV screening as compared to the general population.^20^ HCV screening has been part of a National Hepatitis Screening Program at the VA since 1998, and between 1998 and 2012 the military had much broader recommendations for HCV screening than the national recommendations at that time.^21^ The higher screening rates for patients at a VA contrasted with the low screening prevalence for patients in our sample with military insurance likely indicate that the type of insurance is not limiting, but rather, the location at which the patient received the screening test.

Patients who saw a primary care provider had more than 3 times the odds of receiving HCV screening orders than those who saw specialists only. This association remained significant after adjusting for the total number of visits; therefore, it is not simply a reflection of increased odds of screening due to increased provider encounters. Preventive medicine is a major component of primary care provider practice, compared to specialty care where routine screening recommendations outside of their specialty are unlikely to be addressed.^22^ Data from this study indicate the need and importance of patients having a routine source of health care, particularly primary care services. Additionally, if a patient saw only advanced practice professionals (APPs; eg, nurse practitioners, physician assistants, etc) and not physicians, they had significantly lower odds of screening. This may be because a patient is more likely to see an APP for an acute visit where preventive health maintenance is generally not addressed.^23^

This is among the first studies to use EMR‐verified HCV screening to establish baseline screening prevalence in a large clinic population. The results identify areas for future intervention efforts. However, study findings should be interpreted in light of some limitations. Although our dataset was large and the sample was diverse, it was based on a single healthcare system. We are not able to access patient data outside of our health system so it is possible that the patients in our dataset received HCV screening elsewhere, such as VA facilities in the past. Furthermore, we are not able to assess the reason the provider ordered HCV testing and it is possible some of the HCV testing ordered was for diagnostic purposes, rather than for universal screening. Additionally, we are only able to access screening information after the implementation of the Epic EMR system in August 2015. Patients screened prior to this date were not captured in our dataset. However, our estimates are similar to national data that reported ~12% of baby boomers had been screened by the end of 2015.^10^ Therefore, our results were unlikely to have been significantly affected by this limitation. Additionally, because this is a retrospective review of EMR data, our results are limited by physician recording and proper EMR documentation. However, because this is EMR data, these data are not limited by patient reporting, or recall bias.

While this study does have some limitations, it offers valuable insight into factors associated with patients referred for HCV screening and identifies areas for future intervention efforts. First, our data support the need for patients to have adequate access to primary care. The lower odds of screening among patients who only saw specialists indicate a need for patients to see a primary care provider to ensure screening and increase screening rates in the baby boomer population. Second, future studies could focus on increasing HCV screening during acute visits, increasing screening among women, and emphasizing to providers that baby boomers should be screened for HCV regardless of whether they report risk behaviors. Through continued efforts to increase HCV screening among baby boomers and treat HCV‐related disease across the cascade of care, we have the opportunity to reduce the incidence of HCV‐related morbidity and mortality for a significant proportion of the population.

## CONFLICT OF INTERESTS

ARG is a member of the Merck Advisory Board and currently receives funding through a Merck investigator initiated studies program. STV currently receives funding through a Gilead investigator initiated studies program.

## AUTHORS’ CONTRIBUTIONS

Authors’ contributions are as follows: ML Kasting: Conception and design, interpretation of data, drafting and critically revising article for important intellectual content, approved final version of manuscript to be published. RR Reich: Acquisition of data, analysis and interpretation of data, critically revising the article for content, approved final version of manuscript to be published. LM Duong: Data analysis and interpretation, critically revising the article for important intellectual content, approved final version of manuscript to be published. J Rathwell: Interpretation of data, drafting and critically revising article for important intellectual content, approved final version of manuscript to be published. RG Roetzheim: Acquisition of data, interpretation of data analysis, critically revising article for intellectual content, approved final version of manuscript to be published. AR Giuliano: Conception and design, interpretation of data, critically revising the article for important intellectual content, approved final version of manuscript to be published. ST Vadaparampil: Conception and design, interpretation of data, drafting and critically revising article for important intellectual content, approved final version of manuscript to be published.

## Data Availability

The data that support the findings of this study are available on request from the corresponding author. The data are not publicly available due to privacy or ethical restrictions.
