# Supplementary material for: Electronic medical record‐verified hepatitis C virus screening in a large health system
Source: Cancer Med. 2019 Jun 21;8(10):4555–64. doi: 10.1002/cam4.2247 (PMC6712519; doi:10.1002/cam4.2247)
Supplement: Supplementary file 2 [file CAM4-8-4555-s002.docx]

Appendix B. Factors Associated with Hepatitis C Virus Screening Orders from 2015-2017 for Average Risk Second Youngest Birth Cohort (Born 1966-1985)

|  | Univariate OR  (95% CI) | Multivariable aOR (95% CI) |
| --- | --- | --- |
| ***Born 1966-1985*** |  |  |
|  |  |  |
| ***Characteristics*** |  |  |
| **Age** (Continuous, 5-year increments) | **0.98 (0.97-0.99)** | **0.97 (0.96-0.99)** |
| **Race/Ethnicity** |  |  |
| Non-Hispanic White (ref.) | - | - |
| Non-Hispanic Black | **1.55 (1.30-1.84)** | **1.62 (1.36-1.94)** |
| Non-Hispanic Asian | 1.16 (0.83-1.64) | 1.14 (0.81-1.62) |
| Non-Hispanic Other | 0.81 (0.64-1.02) | 0.91 (0.72-1.15) |
| Hispanic | **1.27 (1.05-1.54)** | **1.30 (1.06-1.60)** |
| **Sex** |  |  |
| Female (ref.) | - | - |
| Male | 0.95 (0.82-1.10) | 0.95 (0.82-1.11) |
| **Language** |  |  |
| English (ref.) | - | - |
| Spanish | 1.05 (0.68-1.62) | 1.04 (0.65-1.66) |
| Other | 0.86 (0.38-1.94) | 0.94 (0.41-2.15) |
| **Payor** |  |  |
| Private (ref.) | - | - |
| Medicaid | **0.48 (0.34-0.67)** | **0.43 (0.31-0.60)** |
| Medicare | 1.05 (0.85-1.31) | 0.98 (0.79-1.23) |
| Medicare Supplement | 1.61 (0.58-4.49) | 1.68 (0.60-4.68) |
| Military | 0.74 (0.51-1.07) | 0.73 (0.50-1.05) |
| Other | **0.47 (0.25-0.88)** | **0.48 (0.25-0.90)** |
| **Total number of healthcare visits** | 1.00 (0.99-1.01) | 0.99 (0.97-1.00) |
| **Type of Visits During Observation Period (7/2015-8/2017)** |  |  |
| Specialty Care Physicians Only (ref.) | - | - |
| Primary Care Physicians Only | **0.67 (0.56-0.80)** | **0.60 (0.50-0.73)** |
| Primary and Specialty Care Physicians | **1.58 (1.20-2.08)** | **1.50 (1.12-2.00)** |
| Advanced Practice Professionals Only | **0.72 (0.59-0.88)** | **0.67 (0.54-0.82)** |
| Unknown | **0.55 (0.35-0.88)** | **0.53 (0.33-0.86)** |
