# Supplementary material for: Electronic medical record‐verified hepatitis C virus screening in a large health system
Source: Cancer Med. 2019 Jun 21;8(10):4555–64. doi: 10.1002/cam4.2247 (PMC6712519; doi:10.1002/cam4.2247)
Supplement: Supplementary file 3 [file CAM4-8-4555-s003.docx]

Appendix C. Factors Associated with Hepatitis C Virus Screening Orders from 2015-2017 for Average Risk Oldest Birth Cohort (Born Before 1945)

|  | Univariate OR  (95% CI) | Multivariable aOR (95% CI) |
| --- | --- | --- |
| ***Born Pre-1945*** |  |  |
|  |  |  |
| ***Characteristics*** |  |  |
| **Age** (Continuous, 5-year increments) | **0.90 (0.86-0.93)** | **0.90 (0.86-0.93)** |
| **Race/Ethnicity** |  |  |
| Non-Hispanic White (ref.) | - | - |
| Non-Hispanic Black | 1.47 (0.78-2.75) | 1.34 (0.71-2.56) |
| Non-Hispanic Asian | **2.51 (1.00-6.25)** | **2.95 (1.15-7.52)** |
| Non-Hispanic Other | 0.70 (0.39-1.24) | 0.93 (0.52-1.67) |
| Hispanic | **1.75 (1.05-2.94)** | 1.59 (0.84-3.01) |
| **Sex** |  |  |
| Female (ref.) | - | - |
| Male | 1.21 (0.89-1.66) | 1.35 (0.98-1.85) |
| **Language** |  |  |
| English (ref.) | - | - |
| Spanish | 1.63 (0.76-3.52) | 1.21 (0.46-3.19) |
| Other | 0.00 (0.00-n/a) | 0.00 (0.00-n/a) |
| **Payor** |  |  |
| Private (ref.) | - | - |
| Medicaid | **4.27 (1.27-14.36)** | **5.02 (1.40-17.96)** |
| Medicare | 1.19 (0.52-2.72) | 1.45 (0.63-3.31) |
| Medicare Supplement | 1.37 (0.48-3.90) | 1.66 (0.58-4.73) |
| Military | 0.00 (0.00-n/a) | 0.00 (0.00-n/a) |
| Other | 0.00 (0.00-n/a) | 0.00 (0.00-n/a) |
| **Total number of healthcare visits** | **1.02 (1.01-1.03)** | 1.01 (0.99-1.02) |
| **Type of Visits During Observation Period (7/2015-8/2017)** |  |  |
| Specialty Care Physicians Only (ref.) | - | - |
| Primary Care Physicians Only | 1.33 (0.87-2.02) | 1.32 (0.86-2.03) |
| Primary and Specialty Care Physicians | **2.51 (1.48-4.27)** | **2.22 (1.27-3.89)** |
| Advanced Practice Professionals Only | **0.36 (0.18-0.72)** | **0.37 (0.18-0.74)** |
| Unknown | 0.87 (0.36-2.11) | 0.82 (0.34-1.99) |
